# Generating Cytokines and Growth Factors for Functional Activity: Feasibility of Method Using MIF Protein

**DOI:** 10.3390/mps7050072

**Published:** 2024-09-12

**Authors:** Hiba Osmani, Ishrya Sharma, Shannon Moonah

**Affiliations:** 1 Department of Medicine, University of Virginia, Charlottesville, VA 22908, USA; hyo5hb@virginia.edu; 2 Department of Medicine, University of Florida, Gainesville, FL 32608, USA; sharmaishrya@ufl.edu

**Keywords:** cytokines, growth factors, human expression system, functional proteins

## Abstract

Cytokines and growth factors are signaling molecules that regulate a variety of biological processes. Understanding their role is essential for basic research and clinical utilization. Thus, cytokines and growth factors are widely used throughout research labs in a significant number of applications. Additionally, genetic polymorphisms result in variant forms of cytokines and growth factors, which can alter their function. Becoming more common, researchers will need to generate these important proteins and their variants themselves in functional forms for activity studies. The expression systems used to generate these proteins can have a major impact on their function. In some instances, post-translational modifications are needed to produce a functionally active protein, which can only be conducted using eukaryotic expression systems. Ideally, for functional relevance, a human expression system should be used for human-related research and applications. Most human cell-based expression systems primarily use HEK (Human Embryonic Kidney) cells; however, relying on just one cell type can lead to several issues, considering the variety of proteins derived from various cell sources. Here, we provide a protocol to effectively and efficiently generate functional recombinant proteins, taking into consideration the diverse range of proteins from different cell types throughout the human body.

## 1. Introduction

Proteins are central to all cell functions. For this reason, advancing current methods for protein expression and purification is essential to enhance our understanding of cellular processes. Cytokines and growth factors are proteins that play important roles in coordinating cellular activities and maintaining tissue function. Disruption of these signaling pathways can lead to various disease conditions, such as cancer and inflammatory disorders [1,2]. Additionally, alterations in the primary amino acid sequence can result in protein variants or isoforms. These changes may lead to altered function, contributing to health and disease. Therefore, understanding the mechanisms and functions of these proteins and their variants is crucial for developing new therapeutic strategies. There are a large number of cytokines and growth factors, with an increasing need for research labs to generate functional cytokines and growth factors themselves.

The expression system used to create recombinant proteins has a significant effect on the resulting product. Some proteins need to undergo post-translational modification for activity. Mammalian cells can perform these modifications, while on many occasions bacteria cannot. For protein expression, selecting a host–cell line that naturally expresses the protein of interest offers several advantages, such as proper folding and certain post-translational modifications that are specifically required for that protein. The majority of expression systems that use human cells rely mainly on one cell type, HEK (Human Embryonic Kidney) cells. This is due to their transfection efficiency and protein production. Relying on a single cell type can have a number of limitations. For example, not all proteins exhibit their native biological activity when expressed in HEK cells, as this cellular environment might lack specific post-translational modifications required for proper function. These modifications, such as glycosylation, can significantly affect protein stability, receptor-binding, and biological functions. For example, interleukin 27 (IL-27), a cytokine belonging to the IL-12 family, is significantly influenced by glycosylation, which plays a crucial role in its biological activity [3]. The Human Protein Atlas is an online resource that provides extensive information on the expression of proteins from a wide range of human cell lines. The cell line section provides the expression profiles of more than one thousand human cell lines [4,5]. This can be used to select a cell line that naturally expresses a protein of interest.

Macrophage migration inhibitory factor (MIF) is a multifunctional protein with both inflammatory cytokine and growth factor functions [6]. Post-translational modifications play crucial roles in MIF protein activity and therefore require a relevant expression system [7,8,9]. Here, we describe a protocol for obtaining functional proteins using MIF as a proof of concept and feasibility of method. 

## 2. Experimental Design

### 2.1. Materials

1. Complete DMEM media: 500 mL DMEM, high glucose (Gibco, Grand Island, NY, USA, Catalog # 11965092), 50 mL (10%) Heat Inactivated Fetal Bovine Serum (Gibco, Grand Island, NY, USA, Catalog # 10082147), 5 mL (1X) Pen/Strep (Gibco, Grand Island, NY, USA, Catalog # 15140122), and 5 mL (1%) Sodium Pyruvate (Gibco, Grand Island, NY, USA, Catalog # 11360070).
2. Fisherbrand™ Surface Treated Sterile Tissue Culture Flasks, Vented Cap (Fisher Healthcare, Houston, TX, USA, Catalog # FB012937).
3. Cell counting slides (Bio-Rad, Hercules, CA, USA, Catalog # 1450015).
4. Trypsin-EDTA (0.05%), phenol red (Gibco, Grand Island, NY, USA, Catalog # 25300054).
5. Opti-MEM™ I Reduced Serum Medium (Gibco, Grand Island, NY, USA, Catalog # 31985070).
6. pcDNA3 Mammalian Expression Vector (Invitrogen, Carlsbad, CA, USA, Catalog # V79020).
7. FuGENE^®^ HD Transfection Reagent (Promega, Madison, WI, USA, Catalog # E2311).
8. Corning™ Costar™ 12-well Clear TC-treated Multiple Well Plates, Individually Wrapped, Sterile (Fisher Healthcare, Houston, TX, USA, Catalog # 07-200-82).
9. Geneticin™ Selective Antibiotic (G418 Sulfate) (50 mg/mL) (Gibco, Grand Island, NY, USA, Catalog # 10131035).
10. Caco-2 cell line (ATCC, Manassas, VA, USA, Catalog # HTB-37^™^).
11. Amicon Ultra centrifugal filter units 30 kDa (Millipore Sigma, Burlington, MA, USA, Catalog # UFC903024).
12. Pierce™ Anti-DYKDDDDK Magnetic Agarose (Thermo Fisher, Waltham, MA, USA, Catalog # A36797).
13. Optional: Pierce™ 3x DYKDDDDK Peptide (Thermo Fisher, Waltham, MA, USA, Catalog # A36805).
14. Wash buffer (DPBS, no calcium, no magnesium, Thermo Fisher, Waltham, MA, USA, Catalog # 14190144).
15. Elution buffer (0.1 M glycine, pH 2.8). Glycine (Millipore Sigma, Burlington, MA, USA, Catalog # G8898).
16. Neutralization buffer (1 M Tris-Base, pH 8). Tris-Base (Millipore Sigma, Burlington, MA, USA, Catalog # T6066).

### 2.2. Equipment

- 37 °C, 5% CO_2_ incubator (Thermo Scientific, Waltham, MA, USA, Catalog # TH-3110).
- Cell culture microscope (Labomed Inc., Culver City, CA, USA, Catalog # S96062).
- Cell counter (Bio-Rad, Hercules, CA, USA, Catalog # 1450102).
- Centrifuge (VWR, Radnor, PA, USA, Catalog # B30316).
- Magnetic separation rack (Cytiva, Marlborough, MA, USA, Catalog # GE28948964).
- Tube rotator at 4 °C (Fisher Scientific, Waltham, MA, USA, Catalog # 88861049).
- Optional: Pulsing vortex mixer (Fisherbrand, Waltham, MA, USA, Catalog # 02-215-422).

## 3. Procedure

### 3.1. Human Cell Line Selection

17. In the Human Protein Atlas database, Version 23, (https://www.proteinatlas.org/, accessed on 19 June 2023), enter MIF into the search bar. We used MIF for our experiments, but any protein of interest can be selected.
18. Under MIF, select your cell line. Here, we used the intestinal cell line Caco2 because it expresses MIF and the cell type was relevant for our work. You can choose other cell lines depending on the nature of the study. The American Type Culture Collection (ATCC) has a collection of more than 4000 cell lines that are available for purchase.

### 3.2. Stable Caco2 Human Cell Line Generation under Antibioitc Selection

19. Count Caco2 cells using an automated cell counter, and, in a 12-well plate, seed 1 × 10^5^ cells per well in 1 mL Complete DMEM media. Note: the media used will be cell type dependent.
20. Incubate at 37 °C.
21. When cells reach 60–70% confluency, transfect Caco-2 cells by adding a 50 µL mixture of pcDNA3 expression vector containing c-terminal FLAG-tagged MIF and FuGENE transfection reagent in Opti-MEM media. The plasmid amount should be 1 µg. FuGENE transfection reagent:plasmid DNA volume ratio should be around 3:1. Add 37 °C prewarmed Opti-MEM media to make up the final 50 µL volume.
22. At 48 h after transfection, aspirate the old media. Add 1 mL of selective media, which is complete DMEM with 400 μg/mL G418, to each well. The pcDNA3 vector contains a neomycin resistance gene that allows for selection with G418. The concentration of G418 may vary depending on the cell type.
23. For the next week, replace the selective media every 2–3 days.
24. When the transfected cells have distinct colonies (cells are growing in patches), split the cells into a tissue culture flask.

### 3.3. FLAG-Tagged MIF Preparation and Protein Purification

25. Grow four tissue culture flasks of Caco-2 cells expressing FLAG-tagged MIF in 15 mL selective media until cells are 70–80% confluent. No additional tags were present in the MIF protein.
26. Collect cell culture media from all four flasks into 50 mL centrifuge tubes. You will have a total of 60 mL of media from the four flasks, so you can put 30 mL into two 50 mL tubes.
27. Centrifuge the media at 3.5 k rpm for 15 min at 4 °C and collect the supernatant with desired secreted protein.
28. Transfer 15 mL of supernatant into an Amicon centrifugal filter unit. An amount of 15 mL is the capacity for the centrifugal filter unit. Minimize the amount of cell debris you add, because it might clog the centrifugal filter unit.
29. Centrifuge at 3.5 k rpm for 15 min at 4 °C.
30. Transfer concentrated media into a 15 mL tube.
31. Repeat steps 25–27 for remaining media (anticipate 3 mL of concentrated media per 15 mL tube; therefore, if you started with four flasks, you should have 12 mL of concentrated media).
32. Add 50 μL of anti-DYKDDDDK magnetic agarose to the 15 mL tube.
33. Incubate the beads overnight while rotating at 4 °C.
34. Place 15 mL tubes on magnetic separation rack so it binds the magnetic beads and then pour off the media without the beads.
35. Resuspend magnetic agarose in 1 mL DPBS and transfer it to 1.5 mL tubes.
36. Wash magnetic agarose with 1 mL wash buffer (DPBS, no calcium, no magnesium). Place tubes on magnetic separation rack and pour off the wash buffer.
37. Repeat step 36 two times for a total of three washes.
38. Incubate the beads in 50 μL elution buffer (0.1 M glycine, pH 2.8) for 2 min at room temperature. Neutralize with 5 μL neutralization buffer (1 M Tris-Base, pH 8).

Alternatives: Instead of eluting with 0.1 M glycine, 3× DYKDDDDK peptide can also be used.

39. Optional: Vortex for 5 min at 1 k rpm.
40. Place 1.5 mL tubes on magnetic separation rack and collect the elute containing the FLAG-tagged MIF in separate 1.5 mL tubes.

## 4. Expected Results

**Figure 1:**
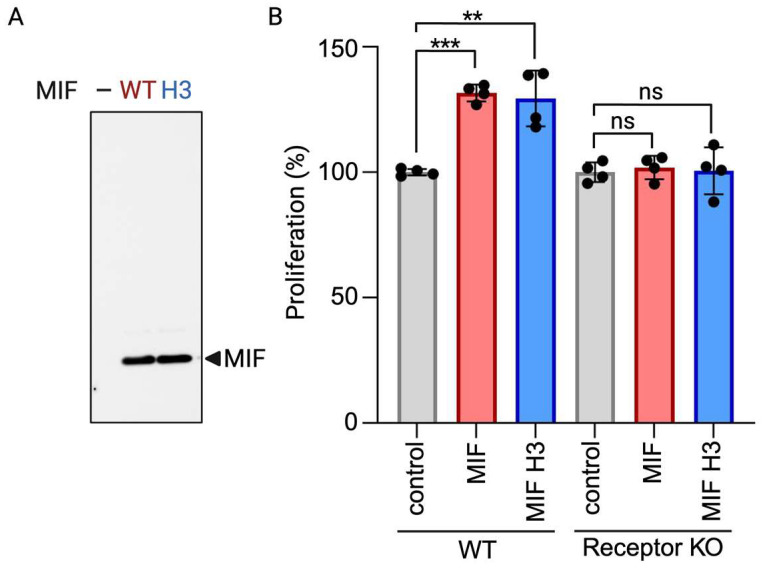
Functional MIF from human cells. (**A**) Immunoblot analysis of MIF expression. (**B**) Percentage proliferation of WT and MIF receptor knockout (CD74 KO) intestinal epithelial cells 24 h after MIF (100 ng/mL) stimulation. Data represent means and SD of quadruplets from 1 experiment and are representative of 3 independent experiments. ** *p* < 0.01, and *** *p* < 0.001.

We successfully yielded one microgram of functional protein per milliliter of media using this purification process. The presence of functional MIF was confirmed through immunoblot analysis and subsequent proliferation assays (Figure 1). It should be noted that the amount of protein obtained after purification depends on the amount of cell media used during the purification process. Some cell types might only express cytokines and growth factors at low levels. In these cases, a higher volume of culture media may be necessary to achieve the desired protein yield. Fortunately, most research studies require only nanogram quantities of cytokines and growth factors [10,11]. While this protocol mainly focuses on production for use in research laboratories, there are cases where large-scale protein production is desired. In such cases, the protocol can be modified using large-scale incubators, often referred to as bioreactors, which facilitate industrial-scale cell culture and protein production, as reviewed in [12].

In this study, we employed a FLAG-tag protein purification strategy to purify MIF. This method is advantageous due to its compatibility with a wide array of expression systems, ease of use, and high specificity. The FLAG-tag system allows for the efficient and specific capture of the target protein, which is essential for downstream applications. By increasing the FLAG-tag length from 1× to 3×, the affinity of the anti-FLAG antibody can be enhanced, likely resulting in a greater yield of purified protein. However, it is important to consider the potential drawbacks associated with using a larger tag. One concern is that the larger tag could interfere with the protein’s native structure and function. This interference could impact the biological activity of the protein, which is important for functional studies. Therefore, while 3× FLAG-tag might improve purification efficiency, careful consideration and optimization are necessary to balance yield with functional integrity.

Optimizing protein yield might also be achieved by adjusting the culture’s temperature. Although mammalian cells are typically cultured at 37 °C to simulate body temperature, lowering the temperature to 33 °C might enhance protein production. Temperature reduction might help to decrease protein aggregation, improve solubility, and potentially optimize post-translational modifications, leading to higher yields of correctly folded and functional proteins. However, the increase in protein production from lowering the temperature will vary depending on the cell line used [13].

## 5. Discussion

In this protocol, we present an efficient method for producing functional recombinant proteins, considering the diverse protein expressions across various human cell types. Proteins like cytokines and growth factors are important for cellular functions, making effective protein expression and purification methods essential for understanding cellular processes. Our approach highlights the importance of selecting appropriate expression systems to ensure accurate protein folding and post-translational modifications, resulting in a higher likelihood of functionality and biological relevance.

Using a cell line that is known to express the protein of interest will significantly increase the chance of expressing a functional protein. For instance, the Chinese hamster ovary (CHO) mammalian cell line is commonly used for protein production. However, when it comes to expressing the growth factor erythropoietin, CHO cells produced erythropoietin with glycosylation patterns that did not closely resemble those of human erythropoietin. In contrast, a human hepatoma cell line (Huh-7) was able to produce erythropoietin in significant levels with proper glycosylation due to its native cellular machinery [14]. The Human Protein Atlas provides an online database that maps human proteins in cells. Resources like the Human Protein Atlas offer useful data on protein expression patterns across various cell lines, which can assist researchers in selecting the best cell lines for their expression studies [4,5]. A potential drawback of using human cell lines is that the desired cell line might not be readily available, for example, through commercial means or collaboration with other research laboratories. One possible solution to overcome this challenge could be to use services that immortalize specific primary cell lines [15,16]. In addition, transfection efficiency can be a challenge, especially when dealing with cell types that are somewhat resistant to traditional transfection methods. That said, the emergence of newer reagents and approaches has provided alternatives for achieving successful transfection results [17,18]. In this protocol, the constitutive cytomegalovirus (CMV) promoter was used in the pcDNA3 vector due to its proven tracking record of success and strong and consistent expression in human cells. Additionally, the CMV promoter has a broad host range, allowing for its use for in a variety of cell types, marking it one of most commonly used constitutive promoters for transgene expression in mammalian cells. The promoter used in the expression vector is critical for maximizing protein yield and activity. Three major promoter types include constitutive, inducible, and native promoters, each containing distinct advantages and challenges that can impact further protein studies. Constitutive promoters drive continuous expression in cells. Commonly used constitutive vectors in mammalian cells include CMV, simian virus 40 early promoter (SV40), chicken beta-actin promoter (CAG), human phosphoglycerate kinase promoter (hPGK), elongation factor 1α promoter (EF1A), and β-Actin promoter [19]. The main advantage of using constitutive promoters is their ability to ensure consistent and reliable expression, which can lead to greater amounts of proteins that may be required for functional assays where a high yield is required. If the protein of interest naturally has a lower yield, then promoters that facilitate high levels of gene expression, such as constitutive promoters, can be used to improve protein yield. However, since constitutive promoters are continuously active, they may overexpress the gene of interest, potentially leading to cell toxicity or stress in cellular machinery, resulting in improper protein folding or post-translational modifications. Inducible promoters allow for precisely regulated gene expression in which molecules termed inducers activate gene expression. Common inducers used in mammalian cells include doxycycline and tetracycline [20]. Inducible promoters allow researchers to express their target protein under regulated conditions. Researchers can control the timing and level of protein expression, allowing for the precise regulation of gene expression and lowering the risks of misfolding or cellular stress due to overexpression associated with constitutive promoters. This may be especially helpful when continuous protein expression causes cellular stress or toxicity.

Expressing proteins from their native human cell lines may uncover additional biological roles and interactions, potentially leading to the discovery of new functions and therapeutic options. Proteins expressed in their original human cells are more likely to have the correct post-translational modifications, including proper folding and glycosylation. Compared to using non-native expression systems, this more closely resembles how things would function naturally within the body and may uncover activities that were otherwise missed. Single amino acid changes can sometimes lead to defects in protein activity. We now know that a significant amount of these proteins are predicted to be pathogenic missense variants that disrupt function [21]. Only a small percentage of variants have been experimentally studied [22]. This protocol will allow researchers to produce not only the wild-type version of proteins, but also their variants. Studying protein variants is required for advancing precision medicine, since it allows for a deeper understanding of how individual genetic differences influence the likelihood of developing diseases and treatment responses. This might ultimately shed light on their impact on essential biological processes, health, and disease.

Overall, this protocol offers an effective and comprehensive approach for producing functional recombinant proteins, enhancing our ability to study cellular processes and develop new therapeutic strategies.

## Data Availability

Data are available on request.
